# The role of MHC genes in contagious cancer: the story of Tasmanian devils

**DOI:** 10.1007/s00251-017-0991-9

**Published:** 2017-07-11

**Authors:** Alison Caldwell, Hannah V. Siddle

**Affiliations:** Department of Biological Science, University of Southampton, Highfield Campus, Southampton, SO17 1BJ UK

**Keywords:** Contagious cancer, Devil Facial Tumour Disease, MHC downregulation, MHC, Vaccine

## Abstract

The Tasmanian devil, a marsupial species endemic to the island of Tasmania, harbours two contagious cancers, Devil Facial Tumour 1 (DFT1) and Devil Facial Tumour 2 (DFT2). These cancers pass between individuals in the population via the direct transfer of tumour cells, resulting in the growth of large tumours around the face and neck of affected animals. While these cancers are rare, a contagious cancer also exists in dogs and five contagious cancers circulate in bivalves. The ability of tumour cells to emerge and transmit in mammals is surprising as these cells are an allograft and should be rejected due to incompatibility between Major Histocompatibility Complex (MHC) genes. As such, considerable research has focused on understanding how DFT1 cells evade the host immune system with particular reference to MHC molecules. This review evaluates the role that MHC class I expression and genotype plays in allowing DFT1 to circumvent histocompatibility barriers in Tasmanian devils. We also examine recent research that suggests that Tasmanian devils can mount an immune response to DFT1 and may form the basis of a protective vaccine against the tumour.

## Introduction

Cells transferred between individuals should be rejected in a robust immune response due primarily to differences in Major Histocompatibility Complex (MHC) genes between individuals (Gorer 1938; Gorer et al. 1948; Snell and Kelton 1953; Dausset et al. 1965; Benacerraf 1992). Tumour cells are no exception, and seminal experiments by Gorer demonstrated that tumour allografts are rejected by host animals (Gorer 1938; Gorer et al. 1948). However, in some species, tumours have emerged that can propagate between individuals, becoming true contagious cancers. The transmission of cancer cells as an allograft in vertebrates is surprising and contradicts our understanding of how histocompatibility barriers function to prevent the transfer of cells between individuals.

In the context of a contagious cancer, cells passing as an allograft should be recognised by T-cells stimulated by allogeneic MHC class I and/or class II molecules present on the cancer cells (Gould and Auchincloss 1999). The stimulation of T-cells by allogeneic MHC molecules is termed direct recognition and initiates a rapid immune response that occurs within 7 to 14 days and results in T-cell infiltration to the graft (Waanders et al. 2007). The transfer of contagious cancer cells should also initiate indirect recognition of the tumour cells where T-cells are stimulated by the presentation of foreign peptides on self antigen presenting cells (APCs) (Dausset 1981; Snell 1981). This process should occur in contagious cancers as cells divide and die in growing tumours, shedding foreign proteins that are taken up by APCs and presented in the context of MHC class II. These foreign peptides could derive from the donor MHC as this is a common event during graft rejection (Benichou et al. 1992). The frequency of T-cells involved in indirect recognition is 100-fold lower than direct recognition but plays a role in chronic graft rejection (Liu et al. 1993). APCs that are transferred to the host along with tumour cells may also play a role where allogeneic MHC molecules are recognised. Transfer of APCs would also introduce MHC molecules from a third individual, as these cells would derive from the most recent host, not the cancer. While this review will focus primarily on MHC-restricted recognition, other mechanisms are also relevant, including host antibodies that could recognise non-MHC antigens on the cancer cells.

**Fig. 1:**
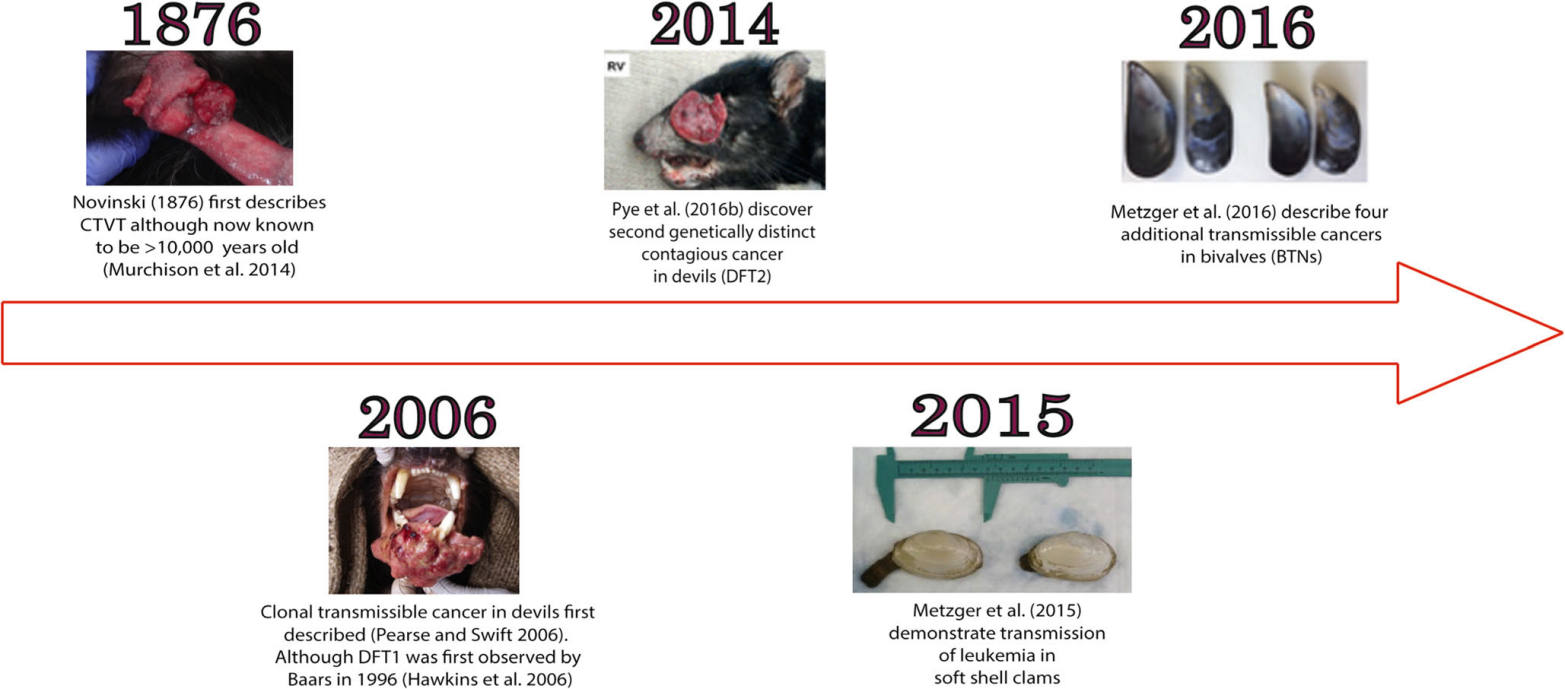
Timeline of the discovery of the contagious cancers CTVT, DFT1, DFT2 and BTNs. The images presented were originally published as follows: CTVT—Siddle et al. (2015); DFT1—Siddle et al. (2015); DFT2—Pye et al. (2016b); clam leukaemia—Metzger et al. (2015); BTNs—Metzger et al. (2016)

Eight naturally occurring transmissible cancers have been found in wild species. In dogs (*Canis lupus*), Tasmanian devils (*Sarcophilus harrisii*) and four species of bivalves (*Cerastoderma edule*, *Polititapes aureus*, *Mytilus trossulus* and *Mya arenaria*), cancer cells are able to pass as an allograft between individuals (Fig. 1). Here we review the transmission of contagious cancers in the Tasmanian devil and the role of MHC class I molecules in this process.

## Examples of contagious cancers

In total, four genetically distinct transmissible cancers have been found in bivalves known collectively as bivalve transmissible neoplasms (BTNs) (Metzger et al. 2015, 2016). The discovery of transmissible cancers in multiple bivalve species in the marine environment, including soft shell clams (*M. arenaria*), mussels (*M. trossulus*), cockles (*C. edule*) and golden carpet shell clams (*P. aureus*), suggests that the transmission of cancer cells may be relatively common among bivalves (Metzger et al. 2015, 2016). These species are filter feeders and transmission experiments have shown that the cancer cells are passed through seawater. Most remarkably, species barriers do not necessarily confine these cancers and the BTN circulating in *P. aureus* derived from a distinct species, *Venerupis corrugata*.

Canine Transmissible Venereal Tumour (CTVT) is a transmissible cancer that arose in an ancient species of dog or wolf more than 10,000 years ago (Novinski 1876; Murchison et al. 2014). CTVT is spread between dogs during coitus and sniffing and licking (Karlson and Mann 1952), with tumours characterised by small, firm, localised nodules around the base of the glans penis of male dogs and in the vaginal vestibulum of female dogs (Murchison 2008). During its long evolution, CTVT has diverged into different genetic subtypes (Murchison et al. 2014) and has spread to all continents, with a higher prevalence in areas where neutering dogs is less common (Strakova and Murchison 2014). With the exception of infection in puppies or in immunocompromised canines, CTVT does not normally metastasize and is rarely fatal (Cohen 1973). The relatively benign nature of CTVT has meant that the tumour has become a form of parasite that co-exists with its host species, the dog.

The Tasmanian devil is a carnivorous marsupial that is endemic to the island of Tasmania south of mainland Australia and is the only mammal in which two genetically distinct contagious cancers have emerged (Pearse and Swift 2006; Pye et al. 2016b). Devil Facial Tumour Disease 1 (DFT1) was first identified in the Tasmanian devil in 1996 (Pearse and Swift 2006), and in 2014, a second transmissible cancer Devil Facial Tumour 2 (DFT2) was discovered (Pye et al. 2016b). Both DFTs cause tumours primarily on the face, neck and oral cavity of the Tasmanian devil, and these tumours are spread through biting during feeding and mating behaviour (Hamede et al. 2008; Murchison 2008). The tumours are first visible as small nodules, usually on a mucosal surface, and after 6 months grow to become multilobed, infected and ulcerated (Loh et al. 2006). DFT1 has a high mortality rate with the first evidence of an immune response to the tumours reported only recently and in only six devils (Pye et al. 2016a). The mortality rate and severity of DFT2 are potentially similar to DFT1, but due to the small number of animals with the tumour, the mortality rate is difficult to determine with confidence (Pye et al. 2016b).

## History and emergence of DFT1 and DFT2

In the 1990s, the Tasmanian devil was relatively common across Tasmania with an estimated population size of 130,000–150,000 devils (McCallum et al. 2007). However, in 1996, a nature photographer, Christo Baars, photographed a Tasmanian devil with a large facial tumour in north-eastern Tasmania (Hawkins et al. 2006). From 1996 to 2001, large facial tumours were observed on devils across the east of Tasmania and the disease was termed Devil Facial Tumour Disease (DFTD now DFT1). At present, DFT1 is present across almost the entire devil range, with only animals in north-west Tasmania disease free (Save the Tasmanian Devil Program 2016). In 2014, a second, genetically distinct contagious tumour, DFT2, was discovered and is thought to be restricted to the south-east of Tasmania (Pye et al. 2016b) (Fig. 1).

DFT1 and DFT2 have a similar gross morphology, but are genetically distinct tumours that most likely originated in different host Tasmanian devils (Pye et al. 2016b). The clonal origin of DFT1 was first proposed due to the highly similar karyotype rearrangements present in tumour samples from different animals (Pearse and Swift 2006). Genetic analysis of DFT1 confirmed that it is a monophyletic clonally transmissible tumour (Siddle et al. 2007; Murchison et al. 2010), and genome sequencing of two geographically distinct DFT1 tumours revealed that it is a relatively stable cell lineage (Murchison et al. 2012). Despite this stability, analysis of 104 DFT1 tumours shows that DFT1 has evolved by linear radiation of subtypes of DFT1 across Tasmania (Murchison et al. 2012).

The evidence that DFT2 is a distinct contagious cancer clone derives from cytogenetics and analysis of microsatellite markers. DFT1 has four unique marker chromosomes not found in host devil cells, and these marker chromosomes are also not found in four DFT2 tumours (Pye et al. 2016b). Further, DFT2 tumours carry a number of other cytogenetic abnormalities in comparison to DFT1 but all have an identical karyotype (Pye et al. 2016b). Interestingly, DFT2 carries a Y chromosome (Pye et al. 2016b) whereas DFT1 is of female origin with no traces of a Y chromosome (Murchison et al. 2012). Furthermore, DFT1 and DFT2 tumours have different genotypes at nine microsatellite markers and the tumours have different MHC genotypes.

## The MHC locus in the Tasmanian devil

There is not yet a comprehensive map of the MHC region in the Tasmanian devil. However, four genomic regions of MHC class I and II genes have been assembled and annotated from Bacterial Artificial Chromosomes (BACs) contigs (Cheng et al. 2012b). All four regions map to chromosome 4q with two regions containing MHC class I genes and two regions containing MHC class II genes (Cheng et al. 2012b). This analysis implies that the organisation of MHC genes in the Tasmanian devil is similar to other marsupials, rather than eutherian mammals, with the MHC class I genes interspersed with genes involved with antigen processing, such as Transporters for Antigen Processing (TAP1 and TAP2) (Cheng et al. 2012b). Three classical MHC class I genes, *Saha-UA*, *Saha*-*UB* and *Saha*-*UC*, and two non-classical MHC class I genes, *Saha-UD* and *Saha-UK*, have been identified on the BACs (Cheng et al. 2012b), and three further non-classical MHC class I genes, *Saha-UM*, *Saha-MR1* and *Saha-CD1*, have been characterised (Cheng and Belov 2014). More recently, a sixth family of non-classical MHC class I genes, the *Saha-UT* family, was identified as a novel family of MHC class I genes unique to non-eutherian mammals (Papenfuss et al. 2015).

**Fig. 2:**
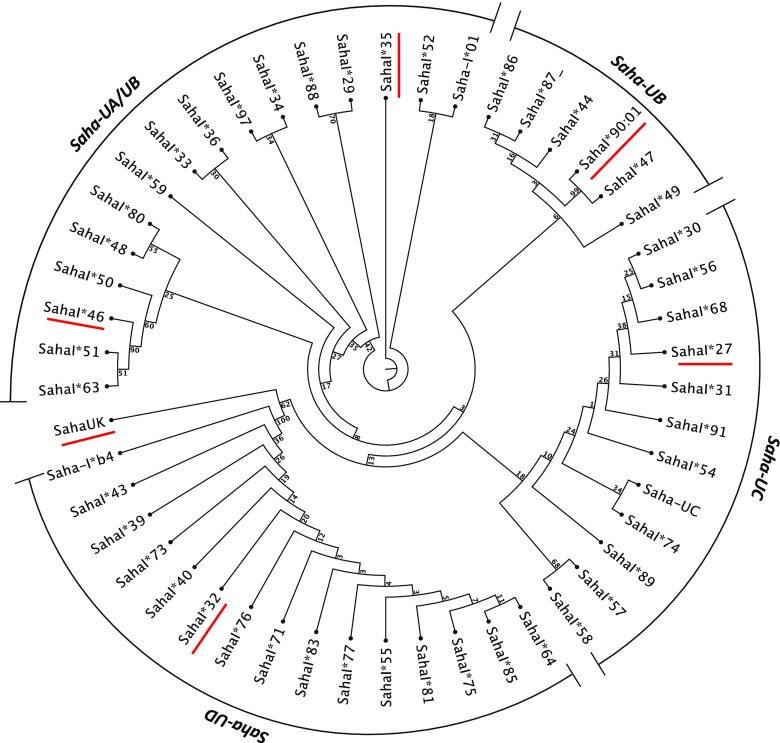
Phylogenetic analysis of exon 2 of the Tasmanian devil MHC class I alleles deposited to the NCBI database. The Neighbour-Joining method with Jukes Cantor distance measurement (1000 bootstraps) was used for the analysis following the phylogenetic analysis of MHC class I in Cheng et al. (2012a). The following MHC class I sequences covering exon 2 of the class I genes were downloaded from NCBI and trimmed to 180 bp of exon 2, EF591089.1; JN397401.1; JN389437.1; JN389435.1; JN389434.1; GQ411488.1; GQ411484.1; GQ411476.1; GQ411466.1; GQ411464.1; GQ411462.1; GQ411460.1;GQ411458.1; GQ411456.1; GQ411452.1; GQ411448.1; GQ411444.1; GQ411442.1;GQ411438.1; GQ411491.1; GQ411493.1; GQ411489.1; GQ411485.1;GQ411483.1; GQ411481.1; GQ411479.1; GQ411471.1; GQ411467.1; GQ411465.1;GQ411463.1; GQ411459.1; GQ411457.1; GQ411455.1; GQ411451.1; GQ411447.1;GQ411441.1; GQ411439.1; GQ411437.1; GU363945.1; GQ411454.1; GQ411435.1; JN389438.1; GQ411443.1; GQ411440.1; KY194696.1; GQ411472.1; KT188437.1; JN397398.1; GQ411482.1. MHC class I sequences were excluded if they did not include sequence for exon 2 of the class I gene. The MHC class I gene of each of the clades has been suggested based on the phylogenetic analysis by Cheng et al. (2012a). The MHC class I alleles expressed by DFT1 are underlined in *red* (colour figure online)

*Saha-UA*, *Saha*-*UB* and *Saha*-*UC* are classified as classical MHC class I genes due to ubiquitous expression and polymorphism in the peptide binding region, but these genes are not orthologous to *HLA-A*, *HLA-B* or *HLA-C* (Cheng et al. 2012b). *Saha-UA*, *Saha*-*UB* and *Saha*-*UC* derive from gene duplications in the devil lineage (Cheng et al. 2012b) and are very closely related genes (Lane et al. 2012). PCR amplification of the MHC class I peptide-binding region of *Saha-UA*, *Saha-UB* and *Saha-UC* identified six *Saha-UA* alleles, seven *Saha-UC* alleles and ten *Saha-UB* alleles (Lane et al. 2012). The MHC class I alleles in the devil are difficult to assign to loci due to their high sequence similarity; this is illustrated by the phylogenetic analysis in Fig. 2.

The class I genes, *Saha-UD, Saha-UK, Saha-UM, Saha-UT, Saha-MR1* and *Saha-CD1*, have been classified as non-classical due to tissue-specific expression, low levels of polymorphism and, in the case of *Saha-MR1* and *Saha-CD1*, orthology to their human counterparts (Cheng and Belov 2014; Papenfuss et al. 2015). *Saha-UD* is expressed in blood, spleen and DFT1 cells and alleles have 97.7% sequence identity in the α1 domain (Cheng et al. 2012b). PCR amplification of the MHC class I peptide-binding region of *Saha-UD* alleles identified four *Saha-UD* alleles (Lane et al. 2012). *Saha-UK* is expressed in the blood and spleen (Cheng et al. 2012b) and is orthologous to a suggested non-classical MHC class I in the closely related marsupials, the grey short-tailed opossum (*Monodelphis domestica*) and tammar wallaby (*Macropus eugenii*) (Siddle et al. 2009). Similarly, *Saha-UM* is orthologous to *-UM* in the opossum and tammar wallaby. The role of the non-classical MHC class I *Saha-UK* and *Saha-UM* is not known, but their conservation between species suggests a marsupial specific function (Siddle et al. 2009; Cheng et al. 2012a). Unlike the other non-classical MHC class I genes, *Saha-MR1* has ubiquitous expression (Cheng and Belov 2014) similar to human *MR1* (Riegert et al. 1998). Although the role of *Saha-MR1* in Tasmanian devils has not been studied, it is expected to play the same role as in humans and activate mucosal-associated invariant T (MAIT) cells (Kjer-Nielson et al. 2012; Cheng and Belov 2014). Similarly, it is suggested that *Saha-CD1* presents lipid antigens during microbial infections (Vincent et al. 2003; Cheng and Belov 2014). There are 13 *UT* family genes located on chromosome 1 in the devil (Papenfuss et al. 2015). Three of these UT genes *UT2*, *UT8* and *UT11* are expressed in spleen but UTs could not be detected in the lymph node (Papenfuss et al. 2015). Given their restricted expression across different tissue types in Tasmanian devils and other marsupials, as well as limited polymorphism, it is likely that the *Saha-UT* genes are involved in roles other than antigenic peptide presentation (Krasnec et al. 2016).

## Transmission of DFT1 and the role of MHC class I molecules

Tasmanian devils have reduced genetic diversity at microsatellite loci (Jones et al. 2007), and their MHC class I and class II genes have fewer alleles than observed in other marsupial species, which has been suggested to play a role in allowing tumour cell transmission (Siddle et al. 2007; Cheng et al. 2012a). The classical MHC class I alleles of *Saha-UA*, and *Saha-UC* share intermediate levels of amino acid identity and *Saha-UB* has 91–99% amino acid identity (Lane et al. 2012). Further, 54% of devils carry a haplotype in which *Saha-UA* is a pseudogene, leaving these animals with only two polymorphic MHC class I genes (Cheng et al. 2012b). Despite low levels of MHC class I diversity in the population, devils can reject skin grafts in a T-cell mediated response (Kreiss et al. 2011) and the level of MHC class I variation in the population should be sufficient to initiate an immune response against DFT1. However, analysis of ten DFT1 biopsies showed poor infiltration of immune cells, including T-cells, B-cells and dendritic cells, suggesting that the devil immune system is ignorant of the tumour (Howson et al. 2014). Interestingly, MHC class II-positive cells have been observed both within the DFT1 tumour and in the stroma, which may represent macrophages in the tumour tissue.

We have shown that the lack of a T-cell response to DFT1 is due to the loss of MHC class I molecules from DFT1 cells. DFT1 cells contain little MHC class I heavy chain molecules and only trace amounts of β_2_m on the cell surface (Siddle et al. 2013). In addition, DFT1 cells do not express MHC class II molecules, but as the cancer derived from a Schwann cell, expression of MHC class II would be unusual. In contrast, Schwann cells in humans and rodents express MHC class I molecules, albeit at low levels, and as such, MHC class I expression would be expected on DFT1 cells (Armati et al. 1990; Meyer Zu Horste et al. 2010). The lack of MHC class I molecules explains the lack of a T-cell response to DFT1 cells, but it does not explain why Natural Killer (NK) cells do not respond to DFT1 due to a missing self ligand.

Loss of MHC class I molecules in DFT1 is due to epigenetic alterations, rather than structural mutations in the DNA (Siddle et al. 2013). The transcripts for β_2_m, TAP1 and TAP2 are downregulated, but some MHC class I heavy chain is still transcribed (Siddle et al. 2013). While there is no evidence of increased methylation at CpG sites in the promoter regions of the β_2_m, TAP1 and TAP2 genes, transcription of these genes can be upregulated when DFT1 cells are treated with the histone deacetylase inhibitor, trichostatin A, which suggests that histone modification, rather than methylation, is involved in MHC class I regulation (Siddle et al. 2013). Further, downregulation of MHC class I expression can be reversed with treatment of DFT1 cells in vitro with interferon gamma (IFNγ), confirming a lack of structural mutations. We have found that MHC class I heavy chain genes, *Saha-UA*, *Saha-UB* and *Saha-UC*, are all upregulated in response to IFNγ treatment, but notably, *Saha-UD* does not respond (Caldwell et al. unpublished). This data fits with the finding that there is no IFNγ response element in the promoter region of *Saha-UD*, in contrast to the other MHC class I heavy chain genes (Cheng et al. 2012b).

Despite the loss of MHC class I molecules from DFT1 cells and high mortality rate among affected devils, there is mounting evidence that the Tasmanian devil immune system is not completely ignorant of DFT1. In addition to the in vitro experiments demonstrating that DFT1 cells upregulate MHC class I in response to IFNγ, DFT1 cells in tumour biopsies have been found that express β_2_m when clusters of CD3-positive leukocytes are adjacent, implying a response to IFNγ or other inflammatory cytokines (Siddle et al. 2013). Importantly, Pye et al. (2016a) have recently identified four wild Tasmanian devils with DFT1 lesions that regressed over time. These animals had serum antibody responses to MHC class I-positive DFT1 cells, but not MHC class I negative DFT1 cells. One of these animals also had CD3-positive lymphocytes infiltrating the tumour tissue prior to regression (Pye et al. 2016a). Taken together, these results indicate that the regression of these tumours was immune mediated and potentially MHC class I restricted. Both MHC class I and class II could be involved in this response as it was not determined whether the DFT1 cells were positive for MHC class II. However, it is possible that serum antibodies against MHC-positive DFT1 cells are not always protective as two additional animals were identified with antibody responses and either MHC class I-positive DFT1 cells in the tumour or evidence of lymphocyte infiltration, but these animals did not show tumour regression at the time that they were captured. These animals were not trapped again, so ultimately, their DFT1 status remains unknown.

The ability of DFT1 cells to respond to inflammatory cytokines has led to efforts to utilise MHC class I-positive DFT1 cells as a vaccine and immunotherapy. Immunisation of devils has been performed using a number of strategies, including the use of sonicated DFT1 cells and frozen/thawed, sonicated or irradiated MHC-positive DFT1 cells with an adjuvant of ISCOMATRIX, Poly I:C and CpG (Tovar et al. 2017). While the antibody responses to these strategies varied, responses were only seen in animals immunised with MHC-positive DFT1 cells (Tovar et al. 2017). Interestingly, despite being immunised with MHC-positive cells, the animals raised antibodies against both MHC-negative and MHC-positive DFT1 cells, implying that once the devil immune system is activated, an antibody response is possible against MHC negative cells. This is supported by a recent study showing that devil mononuclear can be cytotoxic to DFT1 cells in vitro (Brown et al. 2016). The sonication of DFT1 cells used in immunisations may be of particular importance for these responses, increasing the number of antigens to which the host immune system is exposed. Despite the presence of serum antibodies, the immunisations with MHC-positive DFT1 cells are not protective against inoculation with DFT1 cells (Tovar et al. 2017).

The Tasmanian devils immunised in the experiments described above were also used for immunotherapy trials once tumours had developed. Significantly, while immunisation was not protective, the tumours appeared more slowly in the immunised animals than in the control animal (Tovar et al. 2017). Once tumours were palpable, a combination of live or irradiated MHC-positive DFT1 cells and IFNγ was injected into the tumours. These protocols resulted in regression of the tumours in three of six animals. These experiments pose unique challenges in an endangered species where access to animals is restricted, and due to the small number of animals in the study, the results are not definitive, but a number of conclusions can be drawn. First, tumour regression after immunotherapy is dependant on immunisation, as a non-immunised control did not respond to treatment with MHC-positive DFT1 cells once a tumour had formed. Second, the regression of tumours was associated with infiltration of CD3-positive cells into the tumour mass and these cells were dominated by CD8-positive T-cells, suggesting an MHC class I-restricted response.

## Does MHC genotype affect DFT1 transmission?

The immune response to DFT1 cells described above suggests that the MHC genotype of host devils may be relevant to DFT1 progression despite the loss of MHC class I from the cell surface. While no correlation has been found between host MHC genotype and susceptibility or resistance to DFT1 (Lane et al. 2012), the evidence that MHC class I expression can be upregulated on DFT1 cells and the presence of serum antibodies to MHC-positive cells suggests that the MHC genotype could play a role, but is perhaps more subtle than simply resistance or susceptibility. The degree of MHC genotype matching between the tumour and host may contribute to alterations in tumour growth rate and degree of immune response. The MHC class I alleles of the animals used in the immunotherapy trials varied and while no specific links between MHC genotype and response could be made, the variation in response to therapy could be due to genetic background (Tovar et al. 2017).

The MHC class I genotype of DFT1 has not been determined conclusively, and the MHC class I alleles reported in DFT1 cells have varied between studies (Table 1). Pye et al. (2016b) have reported that DFT1 has six MHC class I alleles (SahaI*32, SahaI*35, SahaI*46, SahaI*90, SahaI*45 and SahaI*98), based on sequencing of part of exon 2 of the class I gene from DFT1 biopsies. More recently, it was reported that DFT1 cells have four class I alleles, SahaI*35, SahaI*46, SahaI*90 and SahaI*28 (Tovar et al. 2017). In our own studies of the DFT1 alleles expressed upon stimulation with IFNγ, we find that SahaI*32, SahaI*35, SahaI*46, SahaI*90 and SahaI*28 are all expressed and represent the minimum MHC class I expressed by DFT1 (Siddle et al. unpublished). Although it is difficult to assign these alleles to specific loci, phylogenetic comparison of the DFT1 alleles to all described devil class I sequences suggests that SahaI*35 and SahaI*46 belong to the *Saha-UA* gene and SahaI*90 and SahaI*28 belong to the *Saha-UB* and *Saha-UC* genes, respectively (Fig. 2; Table 1). SahaI*32 is an established allele for the *Saha-UD* gene (Siddle et al. 2007). We also find that *Saha-UK* is expressed by DFT1 cells, but as this gene is not polymorphic, it is likely not relevant in this context. In addition, there is no significant expression of the non-classical MHC class I genes, *Saha-UM* or *CD1* in DFT1 cells (Cheng and Belov 2014).

**Table 1:** MHC class I alleles reported in DFT1 and found to be expressed after treatment with IFNγ

MHC class I gene	Pye et al. (2016b)	Tovar et al. (2017)	Upregulated with IFNγ^*^
Saha-UA	SahaI*35	SahaI*35	Yes
Saha-UA/UB	SahaI*46	SahaI*46	Yes
Saha-UB	SahaI*90	SahaI*90	Yes
Saha-UC	SahaI*27	SahaI*28	Yes
	SahaI*98		Unknown
	SahaI*45		Unknown
Saha-UD	SahaI*32	Not reported	No
Saha-UK	Not reported	Not reported	Yes

Asterisk indicates Siddle unpublished data

## The role of MHC in other contagious cancers

The expression of MHC molecules is also of importance in the progression of CTVT. CTVT has a different pattern of progression to DFT1, once CTVT cells are transmitted, there is a growth period followed by a stationary phase and or regression of the tumour (Cohen 1985). The immune response to CTVT has been reviewed in detail elsewhere (Murchison 2008; Siddle and Kaufman 2015), but it is worth noting that CTVT cells are reported to be MHC class I and class II negative during the growth phase, but become positive during regression of the tumours (Hsiao et al. 2004). While there is no definitive study showing that the immune response to CTVT is MHC class I or class II restricted, the correlation of MHC expression and infiltration of lymphocytes into CTVT suggests that this is the case. In addition, a more dated study, involving inoculation of CTVT into dogs with a degree of MHC matching, suggested that the genetic background of the host dog, including the MHC genotype, is important for the growth of the tumour (Epstein and Bennett 1974). Thus, the level of MHC mismatch may play a role in the spread of this tumour. Indirect support for this hypothesis comes from the suggestion that CTVT emerged during the domestication of dogs, which could have been associated with a rapid decline in genetic diversity among host dogs (Murchison et al. 2014).

There have been a number of isolated cases of tumour cells successfully passing between individuals in humans. These cases have occurred during transplantation and across the maternal/foetal barrier (Isoda et al. 2009; Yagasaki et al. 2011). As for the established contagious cancers in mammals, these tumour cells have needed to overcome at least partial histocompatibility barriers. In the case of maternal to foetus transfer, loss of the non-inherited maternal HLA has been demonstrated (Isoda et al. 2009), while during transplants, there is usually immunosuppression (van Sandwijk et al. 2013). These examples highlight how quickly regulation of MHC molecules can allow transfer of tumour cells.

As invertebrates, bivalves do not have MHC or antigen receptors and phagocytosis is the primary clearance mechanism used to protect against pathogens. However, terrestrial molluscs are capable of recognising differences in cell surface molecules of self and non-self tissue, rejecting allografts through a macrophage and perforin-induced death (Furuta and Yamaguchi 2011), and it is likely that they have a genetic system for the detection of non-self cells, perhaps similar to the system present in colonial chordates.

## Conclusions

The devil immune system does not respond effectively to DFT1 in the majority of cases, as evidenced by the lack of immune cell infiltration (Howson et al. 2014), high mortality rate (Lachish et al. 2011) and vaccination studies (Kreiss et al. 2015). This is due in part to the lack of MHC class I molecules on DFT1 cells, a feature shared with CTVT and many single organism tumours. An outstanding question related to MHC class I loss is why NK cells do not target the tumour cells. However, the regulation of MHC class I expression by epigenetic mechanisms is significant as this type of MHC loss can be reversed using epigenetic modifiers and/or inflammatory cytokines, causing MHC molecules to be returned to the cell surface. Regulation of MHC genes may have an evolutionary benefit to a contagious cancer, allowing the host to survive and transmit tumour cells (Siddle et al. 2013). In DFT1, there is now evidence that the devil immune system is capable of responding to these antigens leading to tumour regression in wild species (Pye et al. 2016a) and after vaccination and immunotherapy with MHC-positive DFT1 cells (Tovar et al. 2017).

When MHC class I molecules are upregulated on DFT1 cells, mismatches in MHC alleles between the tumour and host become relevant. As an allograft, DFTs should provide additional antigenic stimuli to host devils when compared to single organism tumours. In humans, the position and number of mismatches at specific HLA correlates with graft failure (Petersdorf 2017). It is likely that the immune response, or lack of, to DFT1 may also be dependant on specific allelic mismatches between the tumour and the host. Due to recent advances in workflows for identifying tumour-specific antigens, peptide vaccines are under intense scrutiny in human cancers (Yadav et al. 2014; Khodadoust et al. 2017) and these have application in DFT1 where polymorphism between the host devils and the tumour could be exploited to develop a targeted peptide vaccine against the disease. By identifying the mismatches in MHC molecules and bound peptides that determine the ability of host animals to respond to the tumour, we may be able to identify the ‘line’ that allows individuals to distinguish self and non-self.
